# Can Nocturnal Flight Calls of the Migrating Songbird, American Redstart, Encode Sexual Dimorphism and Individual Identity?

**DOI:** 10.1371/journal.pone.0156578

**Published:** 2016-06-10

**Authors:** Emily T. Griffiths, Sara C. Keen, Michael Lanzone, Andrew Farnsworth

**Affiliations:** 1 Bioacoustic Research Program, Cornell Laboratory of Ornithology, Cornell University, Ithaca, New York, United States of America; 2 Powdermill Avian Research Center, Carnegie Museum of Natural History, Pittsburgh, Pennsylvania, United States of America; 3 Information Science, Cornell Laboratory of Ornithology, Cornell University, Ithaca, New York, United States of America; Liverpool John Moores University, UNITED KINGDOM

## Abstract

Bird species often use flight calls to engage in social behavior, for instance maintain group cohesion and to signal individual identity, kin or social associations, or breeding status of the caller. Additional uses also exist, in particular among migrating songbirds for communication during nocturnal migration. However, our understanding of the information that these vocalizations convey is incomplete, especially in nocturnal scenarios. To examine whether information about signaler traits could be encoded in flight calls we quantified several acoustic characteristics from calls of a nocturnally migrating songbird, the American Redstart. We recorded calls from temporarily captured wild specimens during mist-netting at the Powdermill Avian Research Center in Rector, PA. We measured call similarity among and within individuals, genders, and age groups. Calls from the same individual were significantly more similar to one another than to the calls of other individuals, and calls were significantly more similar among individuals of the same sex than between sexes. Flight calls from hatching-year and after hatching-year individuals were not significantly different. Our results suggest that American Redstart flight calls may carry identifiers of gender and individual identity. To our knowledge, this is the first evidence of individuality or sexual dimorphism in the flight calls of a migratory songbird. Furthermore, our results suggest that flight calls may have more explicit functions beyond simple group contact and cohesion. Nocturnal migration may require coordination among numerous individuals, and the use of flight calls to transmit information among intra- and conspecifics could be advantageous. Applying approaches that account for such individual and gender information may enable more advanced research using acoustic monitoring.

## Introduction

The wide array of ecological functions that bioacoustic communication serves is diverse and continually expanding as more studies augment our understanding. Frequently, vocalizations signal membership of a social group or population, or identify individuals among species that form long-term associations with conspecifics (e.g. [1,2,3,4,5]). Among birds, multiple selective and/or ecological forces often shape vocalizations, which, therefore, may encode multiple traits [6,7,8,9]. For example, avian vocalizations frequently signal identifying information about kin or social associations, individual identity, or dominance rank [10,11,12]. This pattern has also been observed in mammals (e.g. vervet monkeys, *Chlorocebus pygerythrus* [13]; sperm whales, *Physeter macrocephalus* [14]; killer whales, *Orcinus orca* [15]; dolphins, *Delphinids* (family)[16], and mechanically in social insects [17,18]. Despite extensive research on how avian vocalizations can encode identifying information about a signaler, most studies have been limited to resident or seasonal breeding populations, or to captive individuals.

Many migrating birds produce species-specific, usually single-syllabled vocalizations that are thought to serve specific functions such as maintenance of flock structure, stimulation of migratory behaviors in conspecifics, and coordination of movements [19,20,21,22,23]. These vocalizations are commonly referred to as nocturnal flight calls, or simply flight calls. These calls may also have utility during winter and post-fledgling periods [24] and there is some evidence of intraspecific variation in flight calls [22,25]. The exact function of calls used in this context is unknown, and no previous studies have examined these signals in a social context, or considered their potential role in signaling traits such as gender, age, or identity.

Unlike animals living in residential social groups or in stable seasonal (breeding and non-breeding) populations, social associations among migrating animals may be temporary and frequently occur among heterospecifics [26,27,28]. Incomplete knowledge of social interactions among individuals during migration, made more difficult by challenges in collecting and processing acoustic recordings of nocturnal flight calls, has resulted in few previous studies of the information that may be present in these calls. Identifying information about a migrant in flight may be valuable for such species with strong habitat and social connectivity, and flight calls may provide a unique and simple source for such information. Therefore, we predict that identifying information may be embedded in these vocal calls.

In this paper we test this prediction by measuring the acoustic features of nocturnal flight calls from migrating American Redstarts, *Setophaga ruticilla*, to determine if flight call similarity can be higher within sexes, age groups, and individual birds, as this could suggest characteristics in calls that convey identifying information. We also analyze call structure in order to identify which features of calls may signal these characteristics. While automated detectors for species identification are currently in development, they are not suitable for widespread use [29]. Relying human experts to classify flight calls to species is still standard methodology for institutions using flight calls to monitor nocturnal migration. Therefore, there is value in determining if there are visual cues that human experts can identify which indicate information of the caller. Establishing qualitative categories to investigate the possibility that particular call variants are more common between genders or among age groups or individuals could help inform conservation and current monitoring techniques.

We chose to study the American Redstart, a long-distance Neotropical migrant that 1) produces stereotyped, species-specific flight calls during migration [22], 2) joins mixed-species passerine flocks, 3) exhibits strong migratory connectivity, with individuals from the same population often migrating together between breeding and non-breeding locations [30,31,32,33], and 4) is strongly sexually dimorphic in many areas of their behavioral ecology, including in plumage, body size [34,35,36,37] and some vocal behaviors [38]. For American Redstarts, difference in gender and age migration is well documented. Older males begin migration before females and younger males to breeding grounds [34,35,39,40]. Males that arrive early increase the likelihood of acquiring a high quality mate and breeding territory [35], and therefore early arrival correlates positively with a larger clutch size and breeding success [40]. Encoding gender and age into migratory flight calls may facilitate or complement this behavior.

## Methods

### Flight call description

**Fig 1:**
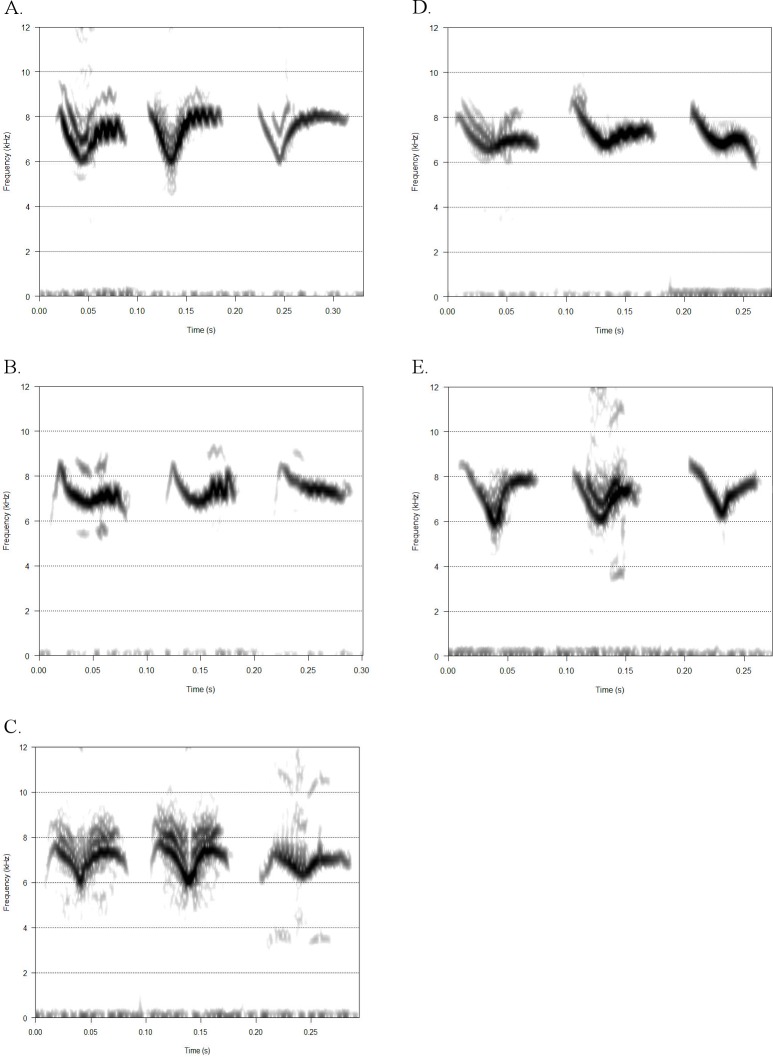
American redstart flight call variants, Hann Window, FFT 256, ovlp = 85%. See supplementary material for more detailed descriptions. a) Type A, a typical redstart flight call as described in Evans and O'Brien (2002); b) Type G, an acute, convex inflection, followed by a shallow, obtuse inflection and modulated tail; c) Type M, two convex inflections at the beginning and tail of call with a concave inflection in the middle; d)Type S, obtuse, concave inflection in middle of call while tail of call is a lower frequency than the beginning; e)Type V, approximately equal in duration on either side of acute concave inflection.

Evans and O'Brien [22] measured free-flying (i.e. wild) American Redstart flight calls to have an average duration of 63.7 ms (range: 49.3–78.8 ms) and ranging from approximately 5.5–10 kHz. Calls generally resemble a 'tick-mark' with a concave inflection followed by a modulated tail (Fig 1). The call starts with a negative slope and inflects into a positive slope. The angle of this inflection can vary (e.g. acute or obtuse). The latter half of the call generally exhibits minimal to heavy modulation, a quick series of inflections with a narrow frequency range (S1 Descriptions). These calls are acoustically and visually distinctive from those of heterospecifics [22,41].

### Data collection

We collected acoustic recordings between September-October 2005 at Powdermill Avian Research Center in Rector, PA from 55 mist-net captured American Redstarts (20 males and 35 females). We conducted work with captive birds in accordance with appropriate Institutional Animal Care and Use Committee (IACUC) protocols in place at and approved by the Powdermill Avian Research Center. We assigned age class assigned to each individual within the guidelines of the USGS age (year class) codes [42] as follows: a Hatch Year (HY) was any bird known to have hatched during the calendar year it was banded; an After Hatch Year (AHY) as any bird older than a HY. Lanzone et al. [25] demonstrated that playback of hetero- and conspecific flight calls often elicited calls from birds held during the banding procedure. They constructed a recording chamber, approximately 30 x 100 cm, to capture evoked flight calls [25]. After banding and measuring each individual to record gender, weight, approximate age (39 HY and 16 AHY) we recorded elicited flight calls from captured birds exposed to playback in the enclosed recording chamber equipped with a Knowles EK3132 microphone with a flat frequency response in the frequency band of interest (5–12 kHz) connected to a computer running Raven Pro 1.4 64-bit [43], which saved all vocalizations produced to 24-bit, 44.1 kHz wav files. Playback calls comprised known flight calls from related, eastern species, played continuously during the temporary capture [25]. We matched the rate of playback calls to those of recordings from typical migration conditions (for examples, see Evans and O’Brien [22]). We released birds after a 10-minute period regardless of the number of calls produced. We collected a total of 776 flight calls (between 1–63 from each individual; mean ± SD: 16.35 ± 13.82).

### Acoustic analysis of calls

We created spectrograms for all recordings using a 256-point Fast Fourier Transform with 256-sample Hann windows and an advance of 38 samples in Raven 1.4. We manually browsed spectrogram using 1.4 s pages with a frequency range of 0–22.5 kHz while simultaneously viewing a plot of sound amplitude versus time displayed above the spectrogram. We drew time and frequency boundaries around all American Redstart flight calls, and we saved these to a Raven selection table. We clipped all selected calls from recordings with 5 ms of padding on either side using SoX (Sound eXchange v. 14.3.1, http://sox.sourceforge.net), resulting in 776 wav files each containing a single flight call. In order to conduct an analysis of call similarity among calls collected from the same individual, in this study we only included birds that produced 5 or more calls during recording (N = 36: 14 males, 22 females; 25 hatching year, 11 after hatching year, Table 1). We randomly selected five calls from each of these 36 individuals, yielding a final dataset of 180 calls. A two-sample ANOVA showed that the means of the feature measurements in the subset did not differ significantly from that of the full dataset (*F =* 9.6, *p =* 0.59).

**Table 1:** Number of birds recorded in both age and gender categories.

Age group	Male	Female	Total
HY	10	15	25
AHY	4	7	11
Total	14	22	36

Age (hatch year = HY, after hatch year = AHY) and Sex break down for all birds used in this study.

For each flight call clip, we measured acoustic characteristics using Raven (i.e. Robust measurements, Raven Pro 1.5; [43]) and Acoustat [44,45,46,47]. These two sets of feature measurements, totaling in 95 variables, quantify the distribution of the power envelop across time and frequency for each clip, and were designed to evaluate regions of the spectrogram containing the most energy (i.e. padding around flight call clips should not affect measurement values). The Acoustat measurements were designed to emphasize distinguishing features of animal sounds, be relatively insensitive to noise interference and temporal artifacts, and yield consistent results despite variation in the shape of the ambient noise power spectra [46,47].In some cases our feature measurements were highly correlated which could skew our final results. To remove this redundancy, we measured the correlation across different feature measurements using the Pearson correlation coefficient. Features that were correlated 95% or more were eliminated from the dataset, leaving 84 variables (S1 Fig). We provide a detailed description of what each measurement is extracting from a flight call clip in S1 Table.

Although all calls in our dataset exhibit similar spectrotemporal structure, we also classified calls visually as one of five different variant classes. We used these qualitative categories to investigate the possibility that particular call variants were more common between genders or among age groups or individuals. Fig 1 shows five flight call variants, with additional detailed descriptions available in the supplementary material.

### Similarity analysis

We applied a permutational multivariate analyses of variance (pMANOVA, described by Anderson [48]) to the remaining feature measurements after the correlation analysis. Individual identity was nested while age, gender, and variant type were applied as fixed factors to ensured that calls from the same bird would not be compared to one another when examining levels of similarity among and within factor groups. Significantly high or low similarity within calls from the same bird did not influence the results of this analysis [49]. We calculated a pMANOVA using 999 iterations with the adonis function in the vegan package (2.3–1, [50]).

To illustrate differences between significantly different qualitative factors, we calculated pairwise similarity scores among all flight calls in our dataset by applying a random forest decision tree (after [51]; randomForest 4.6–12 [52], R version 3.2.2 [53], ntree = 4999) to the feature measurements calculated for each flight call using the default settings. These scores represented the degree of similarity for every call pair, resulting in a 180 x 180 similarity matrix that identified each call’s proximity to its neighbors [52]. Additional random forest analyses were run using similar parameters, only supervised by significant fixed factors. A separate supervised random forest was run per significant fixed factor. Therefore, from the resulting matrices ordination Principal Coordinate Analysis (PCoA) plots of the first two principal coordinates groupings could be generated. This translated the multivariate dispersion relationships within our dataset into a collection of points plotted in a two-dimensional coordinate system.

To determine whether average call similarity was higher among calls from the same individual, we calculated mean similarity among the five calls from a single bird by averaging the 10 pairwise similarity values for these calls, yielding within-individual mean call similarity scores for each 36 individuals. Next, we calculated mean similarity among the five calls from a single individual to all other calls in the dataset (i.e., averaging 5 x 5 x 35 = 875 similarity values from the similarity matrix), yielding 36 between-individual mean similarity scores. We then applied a two-sample t-test to determine whether within-individual mean call similarity was significantly different from between-individual mean call similarity per individual. To visually represent these similarities, we chose two-dimensional non-metric multidimensional scaling (NMDS) plot. Each data point in this plot represents a single flight call, and the distance between every pair of points corresponds to the pairwise similarities of those calls.

## Results

**Fig 2:**
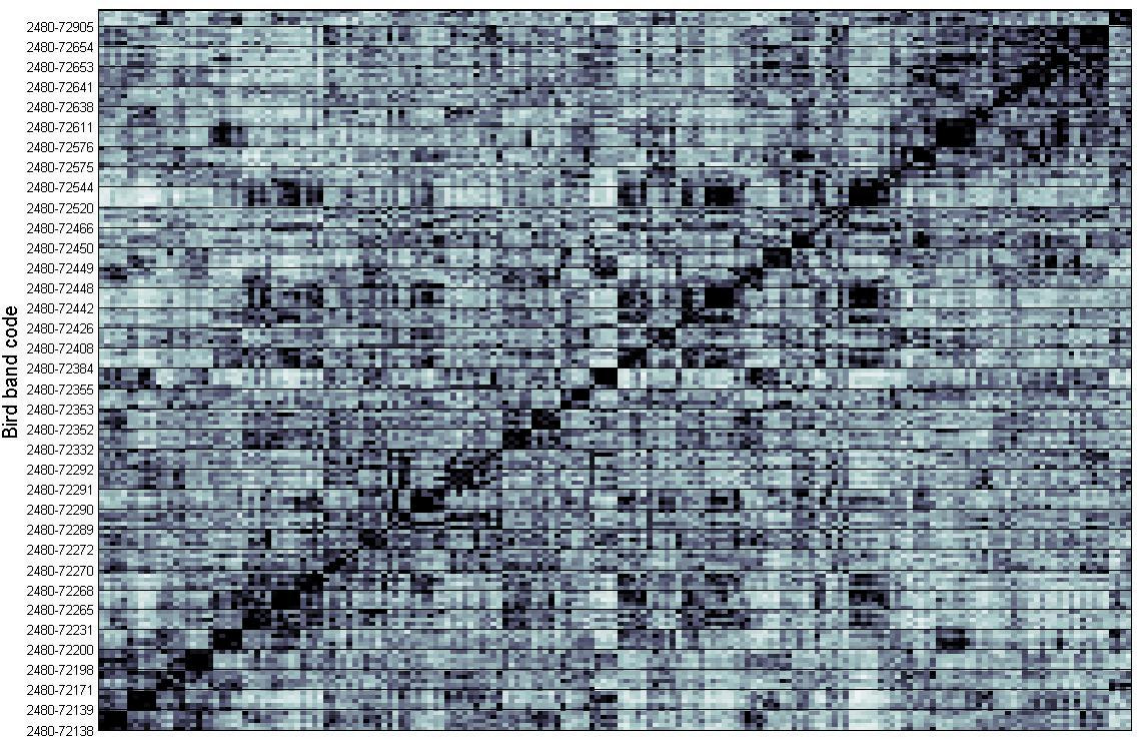
2-D Principal Coordinate Analysis (PCoA) plot based on extracted feature measurements showing multivariate homogeneity of group dispersions between all calls (N = 180),with 99% confidence ellipse based on the standard errors of the axis score averages. Calls from the same sex are plotted in relation to their centroids (M = Male, F = Female). Differences between sexes were shown to be statistically significant (MANOVA; *F* = 12.46, R^2^ = 0.059, *p* < 0.001). Two orthogonal axes summarize the variability in the data set.

Descriptive statistics for American Redstart flight calls were similar to those previously published, with an average duration of 68 ms (range: 38–95 ms) and a frequency range between 3.5–10.3 kHz (S2 Table). Between genders, the mean call duration was 72 ms for males and 66 ms for females, while the frequency range was 3.5–9.8 kHz and 5.2–9.8 kHz for males and females, respectively. For the two age groups, the mean call duration was 68 ms and 69 ms, and the frequency range was 3.5–9.8 kHz and 5.2–9.5 kHz for hatch year (HY) and after hatch year (AHY), respectively. Most calls were variant type A (42.2%), followed by type V (34.1%), S (15.7%), G (4.3%), and M (3.8%) (Table 2; descriptive statistics, S3 Table). Fig 2 shows the similarity matrix generated by calculating random forest distance between all Acoustat quantitative features. Applying permutational MANOVAs to feature measurements (N = 84, after removal of highly correlated variables) showed that calls from birds of different sexes were significantly different (*F* = 12.81, R^2^ = 0.061, *p* < 0.001), revealed no significant difference between calls from birds in different age groups (*F* = 0.57, R^2^ = 0.003, *p* = 0.703), and determined that our subjective variant classes were statistically valid (*F =* 4.39, R^2^ = 0.084, *p*<0.001). When applying multiple fixed factors to the feature measurements, only Sex and Variant paired together just qualified as significant (*F =* 1.96, R^2^ = 0.019, *p =* 0.044).

**Table 2:** Number of calls in each variant class by gender and age of bird.

Call variant	Male		Female		TOTAL
	HY	AHY	HY	AHY	
**A**	23	17	18	17	75
**G**	0	0	5	2	7
**M**	6	0	0	0	6
**S**	1	0	22	6	29
**V**	20	3	30	10	63
**TOTAL**	50	20	75	35	180

The number of calls from each variant class that were collected from each sex and age (HY = hatch year, AHY = after hatch year) group for the subset of data.

**Fig 3:**
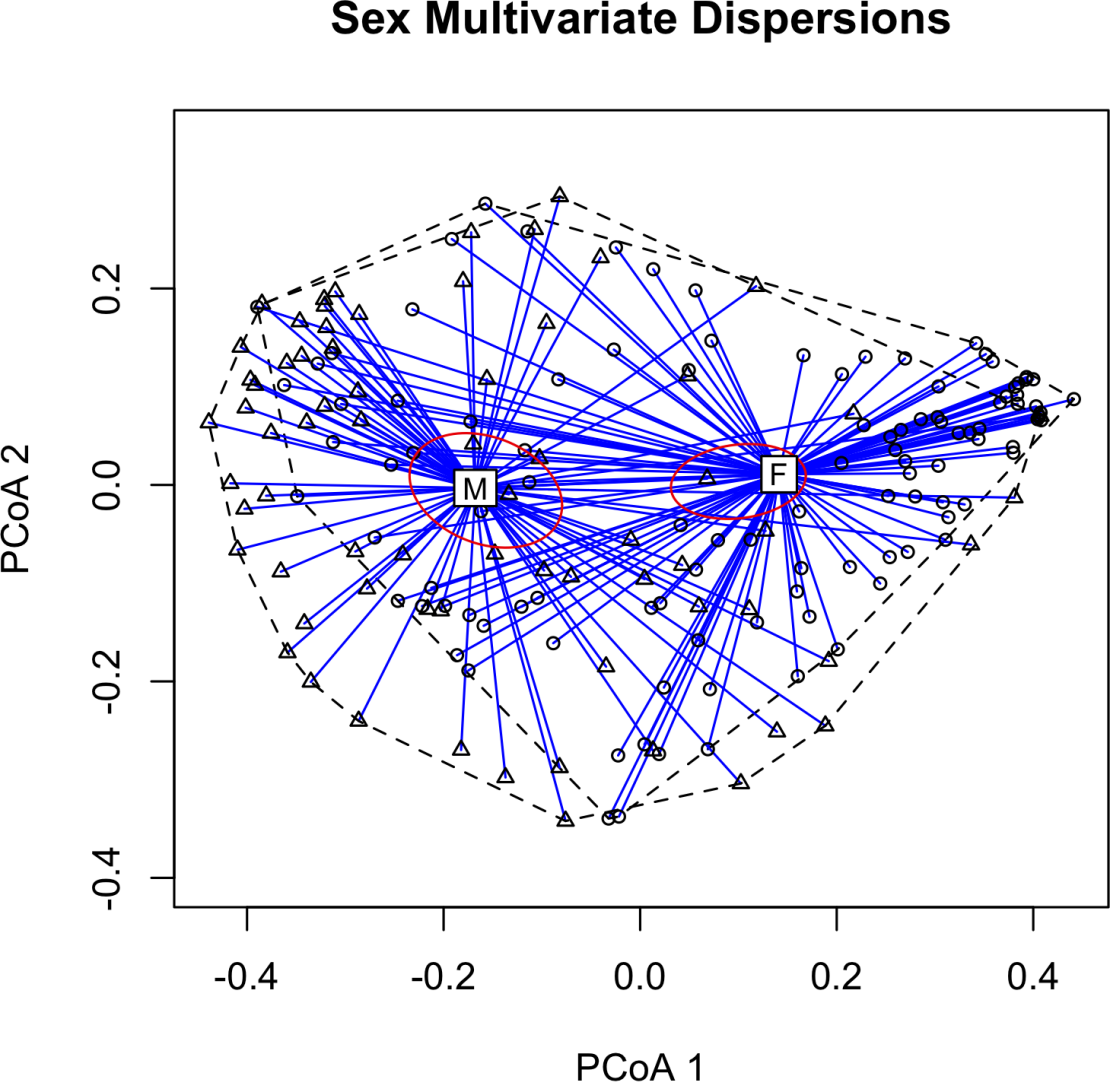
2-D Principal Coordinate Analysis (PCoA) plot based on extracted feature measurements showing multivariate homogeneity of group dispersions between all calls (N = 180), with 99% confidence ellipse based on the standard errors of the axis score averages. Calls from the same variant class are plotted in relation to their centroids. Differences between classes were shown to be statistically significant (MANOVA; *F* = 4.51, R^2^ = 0.086, *p* < 0.001). Two orthogonal axes summarize the variability in the data set. Note: The M centroid is behind the V centroid, but has the large confidence ellipse.

**Fig 4:**
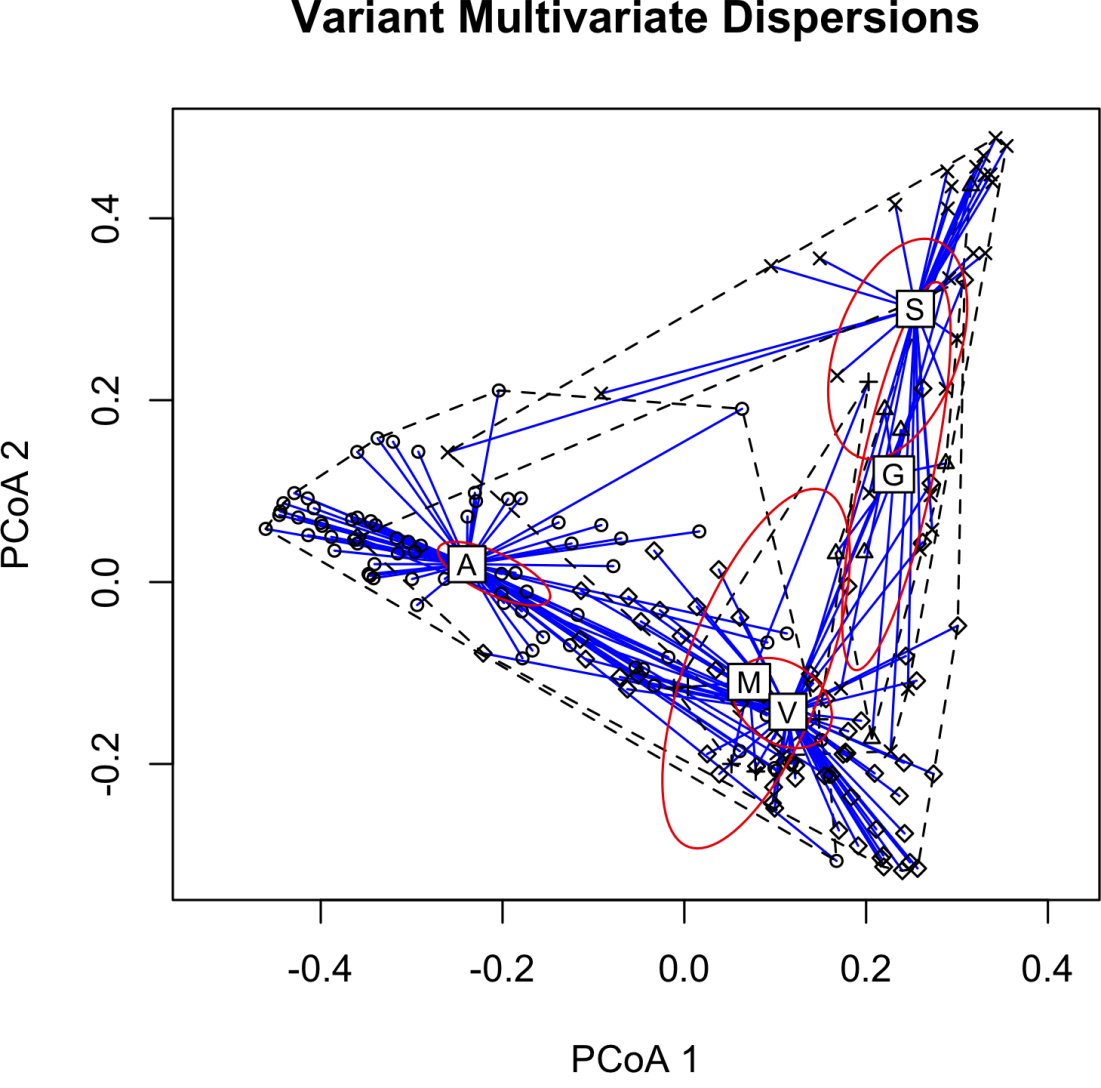
Similarity matrix generated by calculating random forest distance between quantitative features measured from flight calls. Birds are labeled by the bird band assigned given to the bird when the recording was made at the Powdermill bird banding station (S4 Table). The individual pixels in the matrix represent the pairwise similarity values between the 180 flight calls, and the dark grid lines between pixels separate the calls from different individuals (N = 36 individuals, with 5 calls from each). Darker pixels indicate higher pairwise similarity.

PCoA plots showing multivariate group variance relationships between all calls translated into two dimensions and with gender groups and variant classes clustered are shown in Figs 3 and 4. A random forest distance algorithm ranked features not excluded by the correlation analysis as those most useful in calculating similarity between calls for sex (S1 Fig) and variant (S2 Fig). These measurements include, but are not limited to, the time correlation envelope median, the 90% bandwidth, and median frequency contour spread for variant type; the aggregate magnitude spectrum concentration, and the aggregate power spectrum modewidth and concentration for gender. Additionally, comparing within- and between-individual mean call similarity showed that calls from the same individual were significantly more similar to one another than calls from other individuals (*t*-test; *t*_69_ = 12.7, *p* << 0.001, Fig 4, S2 Table). S3 Fig shows a 2-D non-metric multidimensional scaling (NMDS) plot with all calls from the same individual clustered together.

## Discussion

Several recent studies have highlighted the importance and potential of monitoring flight calls of nocturnally migrating birds (e.g. [40,54,55]), but none of these studies or any previous have examined the potential for individual, age, or gender variation in these calls’ spectral and temporal characteristics. Calls collected from the same individual were significantly more similar to one another than to those made by other birds, and call similarity was higher within sexes than between sexes. These results suggest that American Redstart flight calls could encode individual and gender information. Nocturnal migration presumably prevents the use of any visual cues to identify members of a migratory group (e.g. sexually dimorphic plumage or body size), and embedding identifying information in vocal signals may have utility for the signaler or receiver. If there is value in traveling in groups that have stable composition of individuals or gender, or that have intent to travel between the same breeding and non-breeding areas [56,57], flight calls could serve as an ideal medium for communicating this identifying information.

The presence of individual information in flight calls of migratory American Redstarts suggests that social interactions among migratory groups of songbirds may be more complex than previously thought. Individually distinct flight calls could facilitate individual discrimination within migratory groups, a behavior that is not documented during songbird migration but has been observed during seasonal occupancy of breeding or overwintering grounds in several songbird species (e.g. Indigo buntings, *Passerina cyanea* [58]; Hooded warblers, *Setophaga citrina* [59]; Banded wrens, *Thryophilus pleurostictus* [60]; and Song sparrows, *Melospiza melodia* [61]). Since composition of migratory groups is impermanent and likely to change from year to year, American Redstarts may benefit from signaling identity with calls to maintain associations with specific individuals traveling from the same breeding and non-breeding areas [31], rather than any conspecific during migration. Alternatively, individuality in American Redstart flight calls may arise from physiological differences among individuals, irrespective of facilitating caller recognition. Although individuality provides a basis for recognition, such distinction does not demonstrate that true individual recognition occurs [62].

Several social bird species signal gender in their vocalizations (e.g. [12,63]), and such differences in calls have also been observed in the non-social, sexually monomorphic to facilitate sex-specific discrimination at night (e.g. Swinhoe's Storm-Petrel, *Oceanodroma monorhis* [64,65]). There are complex biological and ecological functions which may be associated with broadcasting the gender composition of a flock, and differences in migration behavior between male and female American Redstarts is well documented [34,35,39,66]. In addition to coordinating flock composition by providing cues about migration activity, flight calls could also coordinate a very different suite of behaviors associated with gender, such as triggering competition for breeding resources.

Although age-specific communication may be important for defining migratory behaviors in American Redstarts [34,67,68], flight calls were not significantly more similar within age groups than among age groups. During the breeding season redstarts have some territorial songs that change from year to year, but they also have 'repeat' songs which remain the same [38]. Flight calls could be a vocalization that does not alter as the bird ages. If experienced breeding individuals dictate order of migration (e.g. older male birds migrating before younger males, [66,69]), a signal of age in flight calls may be unnecessary. Moreover, because plumage is a primary indicator of age and reproductive status in American Redstarts [35,70] and visual cues easily communicate breeding status, selective pressures in redstarts may not favor encoding such information in flight calls.

Admittedly, our similarity measurements may not have captured age signaling if it exists. We collected our data from temporarily captive, wild birds, and our recordings could introduce an unforeseen a bias in flight call acoustic structure. However, our measurements of average duration and frequency range are comparable to those collected by Evans and O'Brien [22] on wild birds. Future studies of these behaviors relative to age, sex and individuality could benefit from simultaneous visual identification and flight call recording of free-flying migrants.

The five subjective variant classes for redstart flight calls appear to compose two clusters, A-V-M and S-G. Visually, A-V-M calls have a sharply sloped, acute inflection in the middle of the call, whereas the inflections of S-G calls exhibit an obtuse center inflection. Although both genders produced A and V calls, all G calls and all S calls save one were produced by females. Female callers recorded a shorter duration than males, and call variant S has the shortest duration of the five categories. Only males produced M calls, but this variant type had a small sample size and is highly similar to V calls that, for future studies, the two categories should perhaps be combine into one. These results support that the sex of a redstart may influence the structure of their flight call that human observers can detect. Humans use their natural pattern recognition skills to pick up on diagnostic features, facilitating meaningful signal categorization. In this analysis we identify feature measurements that aid gender and variant classification according to the random forest analysis, though many of these measurements are more useful for future detector and classifier development. The ability to qualitatively and quantitatively assess and monitor migratory flock composition down to gender prediction can be a powerful multifaceted conservation tool.

Due to the challenges in collecting data and in observing and monitoring the nocturnal migration of species directly during large-scale movements, nocturnal flight call research is still in its infancy. Nonetheless, there is great potential for behavioral research and conservation [71], and acoustic monitoring, in particular, has become a valuable tool for using flight calls to identify and discriminate between migrating species [71,72], to examine temporal and seasonal patterns of migration (e.g. [22,23,24,40,73]), and to investigate the evolutionary basis of these vocalizations [74,75]. Regardless of the social function of flight calls during migration, the presence of individual and gender information in flight calls has potential value for furthering such acoustic monitoring applications. Individual discrimination by distinct nocturnal flight calls of different individuals and genders would carry important implications for spatial and temporal monitoring of behavior during migration [76]. Such techniques have been successfully implemented to map territorial occupancy in resident tropical birds [77] and could have utility to understand less complex behavioral patterns, such as group size and composition, or differences in calling behavior among individuals within a group.

Flight calls may serve multiple purposes in during migration, including group cohesion, coordination of movements, and individual and sex-specific discrimination. Continued application of acoustic monitoring techniques and collection of more flight call data will allow a better understanding of migration behavior at the species and individual levels. This application should include the role of vocal communication for coordinating individuals and groups during periods where many important cues may be otherwise absent or confounded (e.g. artificial light confusing migrant birds). Our results highlight the unique context of flight calls as a behavior in which to study the social dynamics of migratory groups and the complex functions of vocal signals.

## Supporting Information

S1 Description Further detailed description of the five redstart flight call variant types (A, G, M, S and V) outlined in this study. (DOC)

S1 Fig Dotchart of variable importance as measured by a Random Forest supervised for gender(N = 88). MeanDecreaseAccuracy: directly measures the impact of each feature on accuracy of the model. This permutes the values of each feature and measures how much the permutation decreases the accuracy of the model. MeanDecreaseGini: For every decision tree, every node is a condition on a single feature designed to split the dataset into two so that similar response values end up in the same set. Using Gini impurity, this measure is based on which the (locally) optimal condition is chosen. Abbreviations of each measurement are explained in S1 Table.
(TIFF)

S2 Fig Dotchart of variable importance as measured by a Random Forest supervised for variant class (N = 88). MeanDecreaseAccuracy: directly measures the impact of each feature on accuracy of the model. This permutes the values of each feature and measures how much the permutation decreases the accuracy of the model. MeanDecreaseGini: For every decision tree, every node is a condition on a single feature designed to split the dataset into two so that similar response values end up in the same set. Using Gini impurity, this measure is based on which the (locally) optimal condition is chosen. Abbreviations of each measurement are explained in S1 Table.
(TIFF)

S3 Fig NMDS plots showing pairwise similarity relationships between all calls (N = 180) within each individual. Calls from the same individual are plotted in the same color and enclosed in centroids. Differences between individuals were shown to be statistically significant (*t*-test; *t*_69_ = 12.7, *p* << 0.001).
(JPG)

S1 Table Descriptions of Acoustat measurements. Description of the Raven Robust measurements come from Charif et al., 2008.
(DOC)

S2 Table Mean and standard deviation Acoustat and Raven Robust feature measurement values for full and subset (N = 5 per individual) dataset. A t-test was run on these means to determine if individual measurements means varied significantly between the two groups. To better describe the subset used in this analysis, measurement minimum and maximum have also been included.
(XLS)

S3 Table Descriptive statistics for the variant classes described in this manuscript and supplementary material. The minimum, maximum, median, mean, and first and third quartiles are presented for the duration, center, low, and high frequency of the five variant classifications: A, G, M, S, and V.
(DOC)

S4 Table Details for the 180 flight calls analyzed in this study, (N = 5 per bird). Presented are the age, sex, and bird band ID of each animal, as well as Raven Robust and Acoustat feature measurements, and variant type for each call.
(CSV)
