# Correction: Can Nocturnal Flight Calls of the Migrating Songbird, American Redstart, Encode Sexual Dimorphism and Individual Identity?

**DOI:** 10.1371/journal.pone.0160596

**Published:** 2016-07-29

**Authors:** Emily T. Griffiths, Sara C. Keen, Michael Lanzone, Andrew Farnsworth

Figure legends for Figs 2–4 are incorrectly aligned. Please view the correct Figs 2–4 and legends here.

**Fig 2:**
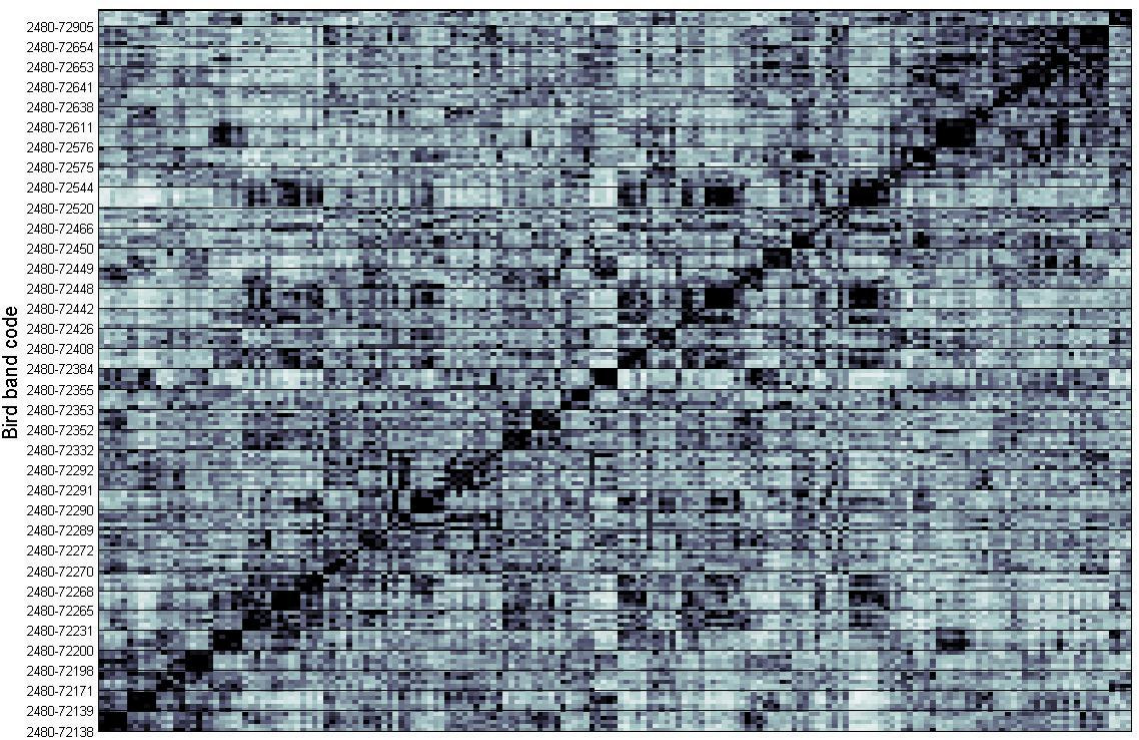
Similarity matrix generated by calculating random forest distance between quantitative features measured from flight calls. Birds are labeled by the bird band assigned given to the bird when the recording was made at the Powdermill bird banding station (S4 Table). The individual pixels in the matrix represent the pairwise similarity values between the 180 flight calls, and the dark grid lines between pixels separate the calls from different individuals (N = 36 individuals, with 5 calls from each). Darker pixels indicate higher pairwise similarity.

**Fig 3:**
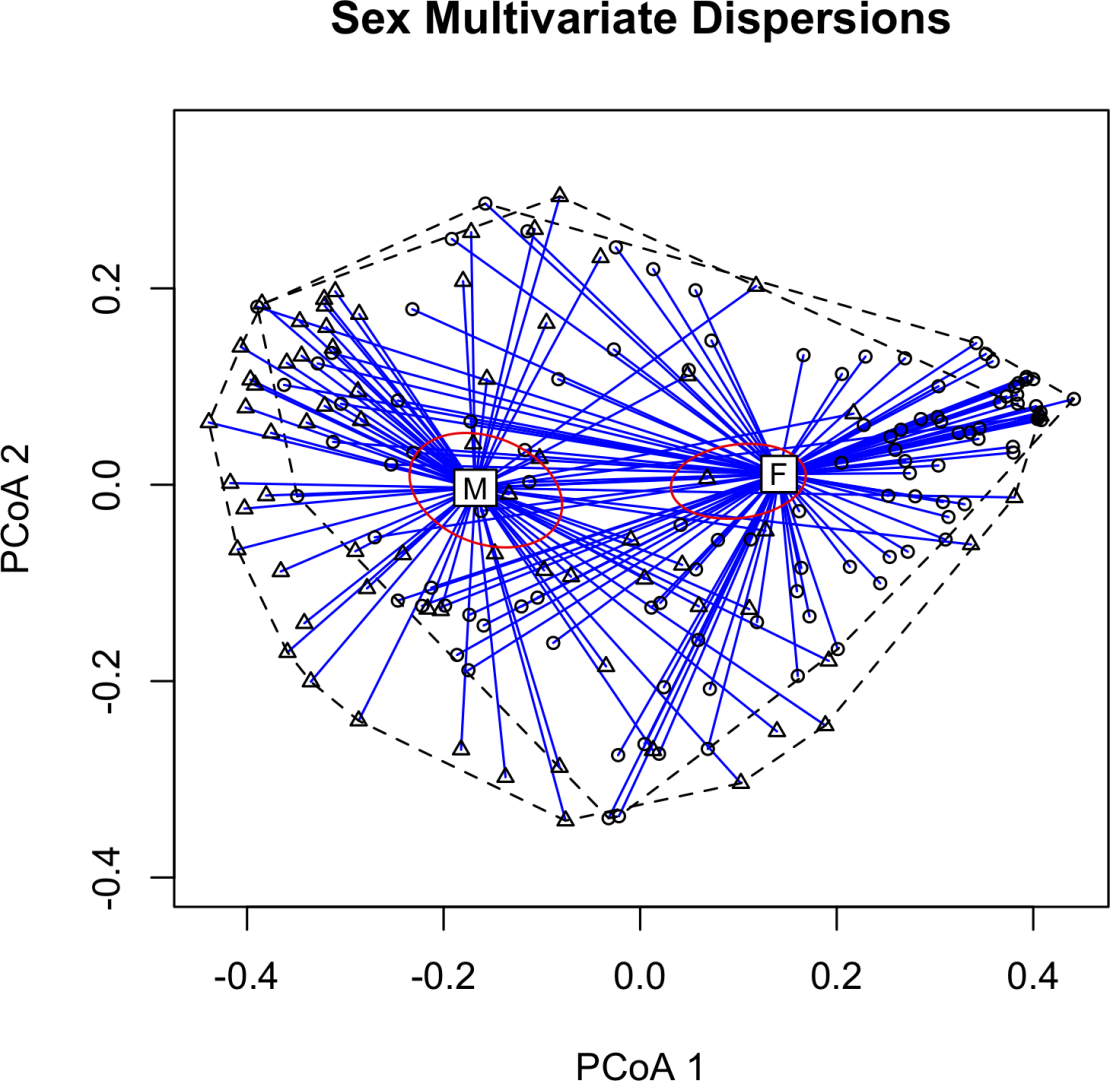
2-D Principal Coordinate Analysis (PCoA) plot based on extracted feature measurements showing multivariate homogeneity of group dispersions between all calls (N = 180), with 99% confidence ellipse based on the standard errors of the axis score averages. Birds are labeled by the bird band assigned given to the bird when the recording was made at the Powdermill bird banding station (S4 Table). The individual pixels in the matrix represent the pairwise similarity values between the 180 flight calls, and the dark grid lines between pixels separate the calls from different individuals (N = 36 individuals, with 5 calls from each). Darker pixels indicate higher pairwise similarity.

**Fig 4:**
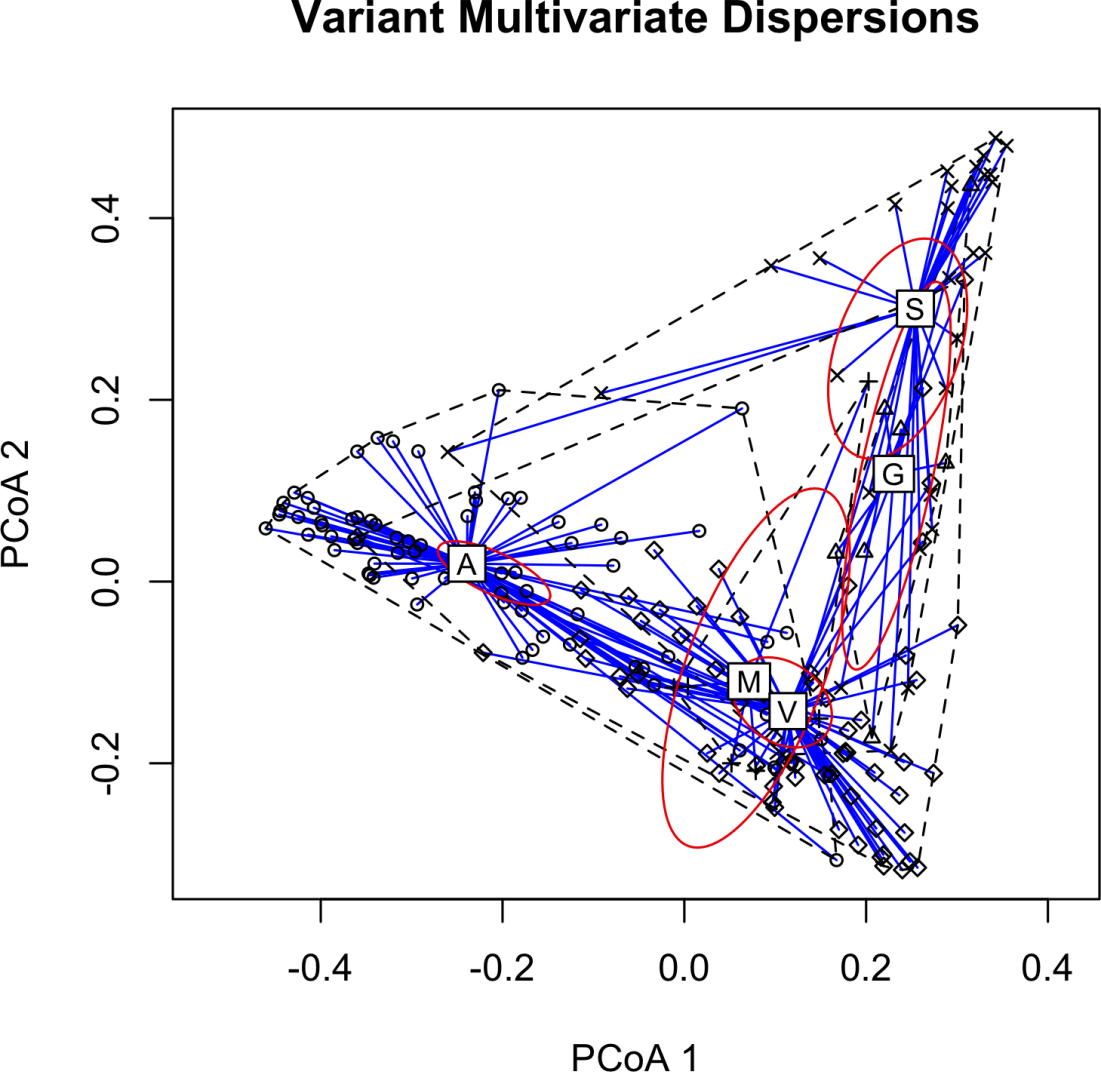
2-D Principal Coordinate Analysis (PCoA) plot based on extracted feature measurements showing multivariate homogeneity of group dispersions between all calls (N = 180), with 99% confidence ellipse based on the standard errors of the axis score averages. Calls from the same variant class are plotted in relation to their centroids. Differences between classes were shown to be statistically significant (MANOVA; *F* = 4.51, R^2^ = 0.086, *p* < 0.001). Two orthogonal axes summarize the variability in the data set. Note: The M centroid is behind the V centroid, but has the large confidence ellipse.
