# Supplementary material for: Can Nocturnal Flight Calls of the Migrating Songbird, American Redstart, Encode Sexual Dimorphism and Individual Identity?
Source: PLoS One. 2016 Jun 10;11(6):e0156578. doi: 10.1371/journal.pone.0156578 (PMC4902225; doi:10.1371/journal.pone.0156578)
Supplement: S1 Description — (DOC) [file pone.0156578.s001.doc]

**Description of flight call variants:**

Although there have been many rigorous studies detailing the acoustic features of flight calls, the automated classification of calls is still in development. Flight calls, by large, can be detected by automated means, but studies still depend on human experts to provide classification [55]. Detailed descriptions of flight call subjective appearance in a spectrogram, in addition to accurate descriptive measurements per species or variant, aid in human identification procedures. The following descriptions are intended for those purposes.

For the purposes of these signal descriptions, concave implies a negative slope inflecting to a positive slope, while convex implies a positive slope inflecting to a negative slope. Modulation implies a compressed section of rapid positive and negative slope inflections creating the illusion of a zigzag when visualized. All AMRE flight calls are comprised of a concave inflection where the angle can vary greatly. It can be an acute or obtuse concave inflection, as in variants A and S, respectively. The latter half of the call generally experiences heavier modulation than the first half, exemplified by variants A and G.

**Variant A:** Standard American Redstart night flight calls as originally categorized by Evans and O’Brien [22]. Resembling a ‘tick mark’, this variant has an acute concave inflection with a heavy modulating tail over a consistent frequency range. Starting at a local frequency maxima for the call, the initial negative slope experiences an acute concave inflection into an positive slope. Once the slope reaches the high frequency boundary again, the call increases in modulation and generally occupies a constant frequency range.

**Variant G:** There is a clear, dramatic positive slope at the start of the call, which leads into an acute convex inflection. This inflection point is the frequency maxima for the call. The negative slope from the convex inflection leads into the main, concave inflection point. The concave inflection point is obtuse, and occasionally absent. Gradually increasing into moderate modulation at a constant frequency, slightly positively sloped, or slightly negatively slope range.

**Variant M:** Though at first glance this variant resembles type A or V, type M has two pronounced local frequency maxima, or two ‘humps’, one on either side of the main concave inflection. This gives the appearance of a cartoon bird in flight; three evenly spaced inflection points. There is a faint, steep, positive slope in the call followed by convex inflection into a negative slope. The call exhibits the main a concave inflection, and the resulting positive slope can experience mild to moderate modulation. A secondary convex inflection into a negative slope may or may not experience a continuum in call modulation.

**Variant S:** A small, downward hook at the beginning of the call turns into a gradual negative slope until the obtuse concave inflection at the center duration. There is a slight positive tendency to the slope in the second half of the call, but the contour generally maintains a constant frequency range while experiencing mild to moderate modulation.

**Variant V**: A short variant, flight call is approximately equal in duration on either side of the acute concave inflection point. In appearance, it resembles the letter “V”.
