# Supplementary figures and images for: Can Nocturnal Flight Calls of the Migrating Songbird, American Redstart, Encode Sexual Dimorphism and Individual Identity?
Source: PLoS One. 2016 Jun 10;11(6):e0156578. doi: 10.1371/journal.pone.0156578 (PMC4902225; doi:10.1371/journal.pone.0156578)

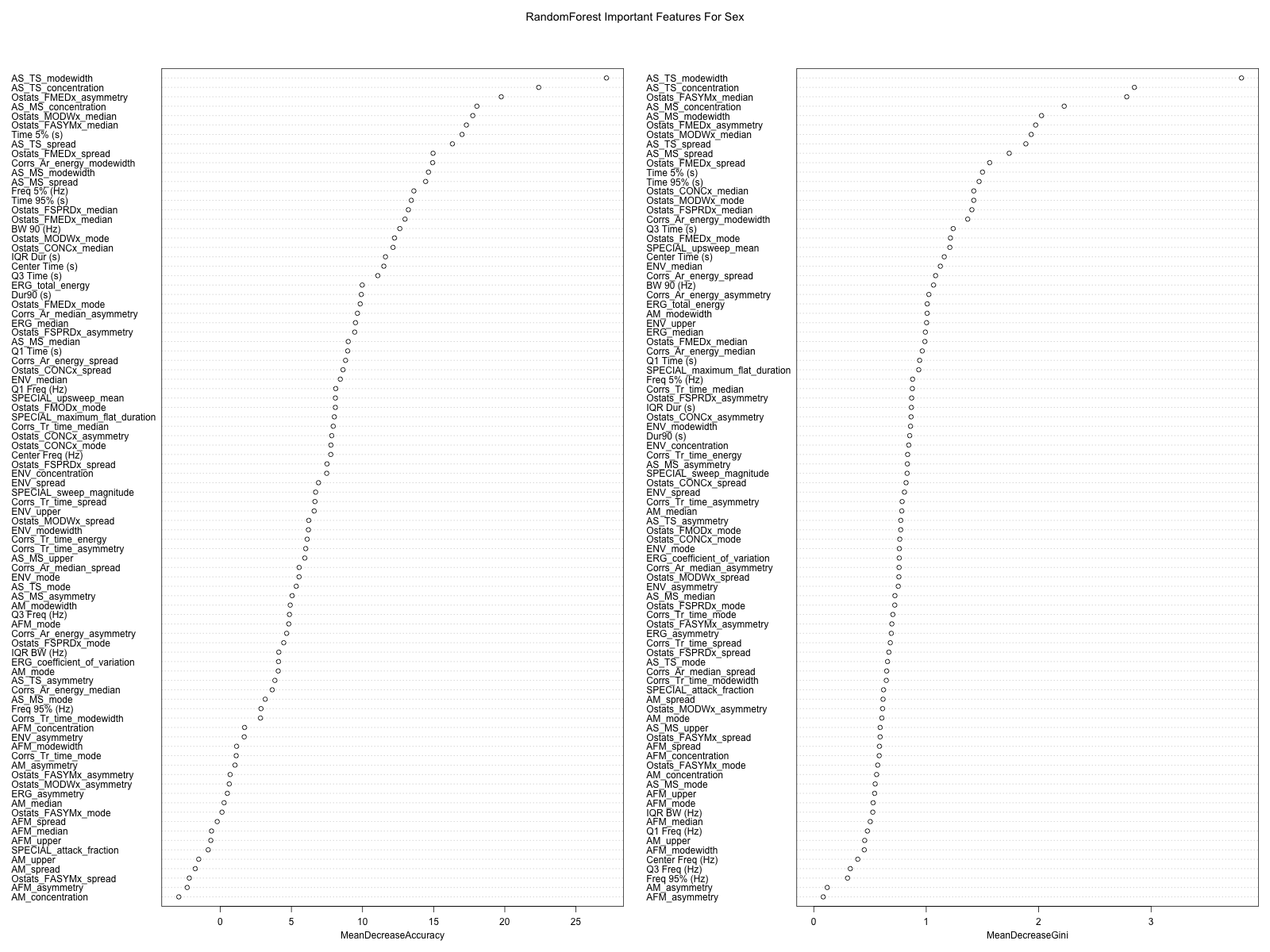

Supplement: S1 Fig — This permutes the values of each feature and measures how much the permutation decreases the accuracy of the model. MeanDecreaseGini: For every decision tree, every node is a condition on a single feature designed to split the dataset into two so that similar response values end up in the same set. Using Gini impurity, this measure is based on which the (locally) optimal condition is chosen. Abbreviations of each measurement are explained in S1 Table. (TIFF) [file pone.0156578.s002.tiff]

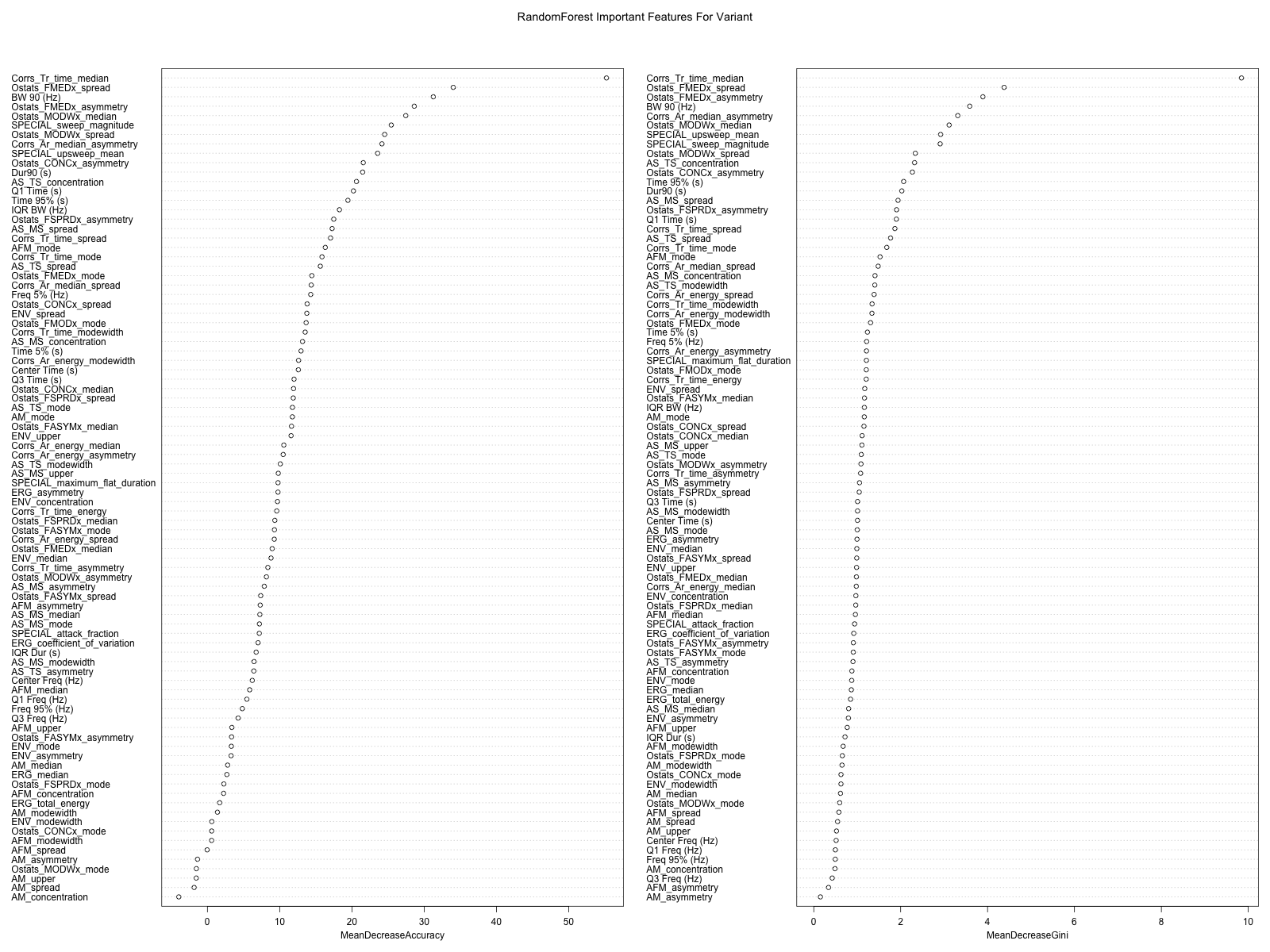

Supplement: S2 Fig — MeanDecreaseAccuracy: directly measures the impact of each feature on accuracy of the model. This permutes the values of each feature and measures how much the permutation decreases the accuracy of the model. MeanDecreaseGini: For every decision tree, every node is a condition on a single feature designed to split the dataset into two so that similar response values end up in the same set. Using Gini impurity, this measure is based on which the (locally) optimal condition is chosen. Abbreviations of each measurement are explained in S1 Table. (TIFF) [file pone.0156578.s003.tiff]

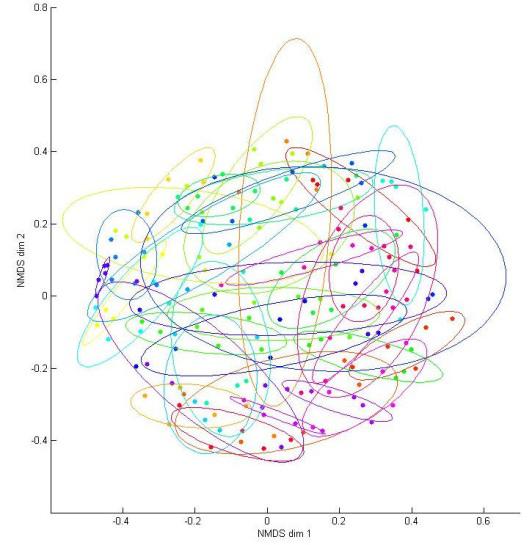

Supplement: S3 Fig — Calls from the same individual are plotted in the same color and enclosed in centroids. Differences between individuals were shown to be statistically significant (t-test; t69 = 12.7, p << 0.001). (JPG) [file pone.0156578.s004.jpg]
