# Supplementary material for: Can Nocturnal Flight Calls of the Migrating Songbird, American Redstart, Encode Sexual Dimorphism and Individual Identity?
Source: PLoS One. 2016 Jun 10;11(6):e0156578. doi: 10.1371/journal.pone.0156578 (PMC4902225; doi:10.1371/journal.pone.0156578)
Supplement: S1 Table — Description of the Raven Robust measurements come from Charif et al., 2008. (DOC) [file pone.0156578.s005.doc]

S1 Table. Descriptions of Acoustat measurements. Description of the Raven Robust measurements are reprinted from Charif et al., 2008.

| ACOUSTAT |  |
| --- | --- |
| Measurement name | Description |
| Aggregate energy envelope (ENV) | Median, mode, upper limit, concentration, modewidth, skewness, and equivalent-duration of the aggregate energy envelope (limits of the signal). |
| Aggregate power spectrum (AS_TS) | Median, mode, upper limit, concentration, modewidth, skewness, and equivalent-bandwidth of the aggregate power spectrum. |
| Amplitude-time envelope (AM) | Median, mode, upper limit, concentration, modewidth, skewness, and equivalent-duration of the amplitude-time envelope (limits of amplitude-time). |
| DTF (AFM) | Median, mode, upper limit, concentration, modewidth, skewness, and equivalent-width of the discrete-time Fourier Transform (DFT) of the median frequency contour from the amplitude-time envelope weighted by ¼ power of the amplitude-time envelope. |
| Aggregate magnitude spectrum (AS_MS) | Median, mode, upper limit, concentration, modewidth, skewness, and equivalent-width of the aggregate magnitude spectrum. |
| Energy distribution (ERG) | Median, coefficient of variation, max over median, skewness, and total energy of energy distribution. |
| Amplitude correlation envelope (Corrs_Ar) | Temporal mode, median, energy, modewidth, spread, and skewness of amplitude correlation envelope (limits of correlation). |
| Time correlation envelope (Corrs_Tr) | Temporal mode, median, energy, modewidth, spread, and skewness of time correlation envelope (limits of correlation). |
| SPECIAL__attack_fraction | Fraction of data blocks that have higher energy than the previous block. |
| SPECIAL__upsweep_fraction | Fraction of data blocks that have higher frequency (based on the peak frequency contour) than the previous block. |
| SPECIAL__upsweep_mean | Average slope of the peak frequency contour. |
| SPECIAL__sweep_magnitude | Sum of the absolute value of the derivative of the peak frequency contour. |
| SPECIAL_maximum_flat_duration | Duration of the longest period during which the frequency contour has a slope of zero. |
| Modal frequency contour  (Ostats_FMODx) | Mode, median, spread, and skewness of the modal frequency contour. |
| Median frequency contour  (Ostats__FMEDx) | Mode, median, spread, and skewness of the median-frequency contour, where FMED is the vector of the median-frequency values in each spectral frame of the power spectrum. |
| Frequency-concentration contour  (Ostats__CONCx) | Mode, median, spread, and skewness of the frequency-concentration contour, where FCC is the vector of the number of bins needed to accumulate 50% of the total energy in the sorted energy distribution for each spectral frame |
| Equivalent-bandwidth contour  (Ostats__MODWx) | Mode, median, spread, and skewness of the equivalent-bandwidth contour, where EBC is the vector of equivalent-bandwidth values for each spectral frame |
| Frequency-spread contour  (Ostats_FSPRDx) | Mode, median, spread, and skewness of the frequency-spread contour, where FSPRD is the vector containing the range of values for each spectral frame. |
| Frequency-skewness contour  (Ostats__FASYMx) | Mode, median, spread, and skewness of the frequency-skewness contour, where FSKEW is the vector of skewness values for each spectral frame |
| RAVEN ROBUST |  |
| Measurement name | Description |
| Center Frequency (CenterFreq) | The frequency that divides the selection into two frequency intervals of equal energy. |
| 1^st^ and 3^rd^ Quartile Frequency (Q1-& Q3Freq) | The frequency that divides the selection into two frequency intervals containing 25% and 75% of the energy in the selection. |
| IQR (Inter-quartile Range) Bandwidth (IQR_BW) | The difference between the 1st and 3rd Quartile Frequencies. |
| Frequency 5% and 95% (Freq5 & 95) | The frequency that divides the selection into two frequency intervals containing 5% and 95% |
| Bandwidth 90% (BW90) | The difference between the 5% and 95% frequencies. |
| Center Time (CenterTime) | The point in time at which the selection is divided into two time intervals of equal energy. |
| 1^st^ and 3^rd^ Quartile Time (Q1-& Q3Time) | The time that divides the selection into two time intervals containing 25% and 75% of the energy in the selection. |
| IQR (Inter-quartile Range) Duration (IQR_DUR) | The difference between the 1st and 3rd Quartile times. |
| Time 5% and 95% (Time5 & 95) | The time that divides the selection into two time intervals containing 5% and 95% |
| Duration 90% (Dur90) | The difference between the 5% and 95% times. |
