# Supplementary material for: Can Nocturnal Flight Calls of the Migrating Songbird, American Redstart, Encode Sexual Dimorphism and Individual Identity?
Source: PLoS One. 2016 Jun 10;11(6):e0156578. doi: 10.1371/journal.pone.0156578 (PMC4902225; doi:10.1371/journal.pone.0156578)
Supplement: S3 Table — The minimum, maximum, median, mean, and first and third quartiles are presented for the duration, center, low, and high frequency of the five variant classifications: A, G, M, S, and V. (DOC) [file pone.0156578.s007.doc]

| **Variant** | **Feature Measurement** | **Minimum** | **1st Quarter** | **Median** | **Mean** | **3rd Quarter** | **Maximum** |
| --- | --- | --- | --- | --- | --- | --- | --- |
| A | Duration (s) | 0.053 | 0.067 | 0.072 | 0.073 | 0.078 | 0.095 |
|  | Center Frequency (Hz) | 5685 | 7063 | 7235 | 7203 | 7407 | 7924 |
|  | Low Frequency (Hz) | 3537 | 5753 | 5966 | 5863 | 6256 | 7034 |
|  | High Frequency (Hz) | 7491 | 8459 | 8667 | 8679 | 8923 | 9531 |
| G | Duration (s) | 0.061 | 0.066 | 0.067 | 0.068 | 0.071 | 0.074 |
|  | Center Frequency (Hz) | 7063 | 7149 | 7752 | 7481 | 7752 | 7752 |
|  | Low Frequency (Hz) | 5324 | 5924 | 6062 | 6115 | 6389 | 6794 |
|  | High Frequency (Hz) | 8511 | 8582 | 8670 | 8669 | 8734 | 8873 |
| M | Duration (s) | 0.069 | 0.074 | 0.076 | 0.076 | 0.080 | 0.083 |
|  | Center Frequency (Hz) | 6546 | 6977 | 7235 | 7120 | 7364 | 7407 |
|  | Low Frequency (Hz) | 5451 | 5726 | 5948 | 5929 | 6172 | 6325 |
|  | High Frequency (Hz) | 8088 | 8122 | 8286 | 8669 | 9183 | 9804 |
| S | Duration (s) | 0.043 | 0.054 | 0.061 | 0.064 | 0.075 | 0.083 |
|  | Center Frequency (Hz) | 6718 | 6891 | 7235 | 7205 | 7580 | 7752 |
|  | Low Frequency (Hz) | 5502 | 6136 | 6414 | 6425 | 6735 | 7164 |
|  | High Frequency (Hz) | 7656 | 8272 | 8493 | 8497 | 8667 | 9484 |
| V | Duration (s) | 0.051 | 0.059 | 0.064 | 0.065 | 0.070 | 0.081 |
|  | Center Frequency (Hz) | 6202 | 6891 | 7235 | 7180 | 7407 | 7924 |
|  | Low Frequency (Hz) | 4642 | 6048 | 6378 | 6243 | 6566 | 6862 |
|  | High Frequency (Hz) | 7701 | 8512 | 8726 | 8686 | 8920 | 9835 |
